# Development of the Near Vision Presbyopia Task-based Questionnaire for use in evaluating the impact of presbyopia

**DOI:** 10.1186/s41687-021-00378-y

**Published:** 2021-12-02

**Authors:** Elaheh Shirneshan, Cheryl D. Coon, Nathan Johnson, Jonathan Stokes, Ted Wells, J. Jason Lundy, David A. Andrae, Christopher J. Evans, Joanna Campbell

**Affiliations:** 1 Allergan, an AbbVie company, 2525 Dupont Drive, T2-2P, Irvine, CA 92629 USA; 2 Outcometrix, St. Petersburg, FL USA; 3 Endpoint Outcomes, Long Beach, CA USA; 4 Allergan, an AbbVie company, Madison, NJ USA; 5 Endpoint Outcomes, Boston, MA USA

**Keywords:** Presbyopia, Patient-reported outcome, Qualitative research, Content-validity, Psychometric analysis, Age-related farsightedness

## Abstract

**Background:**

Presbyopia is a progressive condition that reduces the eye’s ability to focus on near objects with increasing age. After a systematic literature review identified no existing presbyopia-specific patient-reported outcome (PRO) instruments meeting regulatory guidance, a new PRO instrument, the Near Vision Presbyopia Task-based Questionnaire (NVPTQ), was developed.

**Results:**

To explore the patient experience with presbyopia, concept elicitation interviews were conducted with 20 presbyopic participants. The most frequently reported impacts were difficulty with reading menus/books/newspapers/magazines, reading on a cell phone/caller ID, and reading small print. Based on these results, a task-based PRO instrument (the NVPTQ) was developed instructing participants to complete four near-vision, paper-based reading tasks (book, newspaper, nutrition label, menu) under standardized settings, and subsequently assess their vision-related reading ability and associated satisfaction. The draft NVPTQ was cognitively debriefed with a sample of 20 presbyopes, which demonstrated that most participants interpreted the items as intended and endorsed the relevance of the concepts being assessed. After the qualitative research, the draft instrument was psychometrically tested using data from a Phase 2 study. Based on item-level analyses, all items in the NVPTQ demonstrated expected response option patterns and lacked substantial floor or ceiling effects. The reliability, validity, and responsiveness of the NVPTQ Performance and Satisfaction domain scores were assessed. All domains scores had large Cronbach’s coefficient α values and good test–retest statistics, indicating that the scores are internally consistent and produce stable values over time. The pattern of correlations with a concurrent measure of visual functioning (National Eye Institute Visual Function Questionnaire 25) demonstrated that the NVPTQ domain scores were related to an alternative assessment of near-vision activities. The NVPTQ domain scores were able to distinguish between groups that were known to differ on the clinical outcome of uncorrected near visual acuity, supporting the construct validity of these scores. The NVPTQ domain scores showed evidence of responsiveness to change by being able to distinguish between groups defined as improved and not improved based on patient-reported and clinical outcomes.

**Conclusions:**

This research has resulted in a content-valid and psychometrically sound instrument designed to evaluate vision-related reading ability and satisfaction with vision-related reading ability.

*Trial registration*: ClinicalTrials.gov NCT02780115. Registered 23 May 2016, https://www.clinicaltrials.gov/ct2/show/NCT02780115?term=NCT02780115&draw=2&rank=1.

## Background

Presbyopia is an age-related progressive visual condition characterized by the loss of the ability of the eye to focus on near objects. Uncorrected presbyopia has been found to significantly reduce quality of life, as individuals experience a reduction in near visual acuity [1]. The ability to perform common near-vision reading tasks resulting from blurred near vision was identified as an integral presbyopia concept for measurement based on a review of the literature [2]. To manage the impacts of presbyopia, individuals often implement various coping mechanisms to better see things at close distance, such as squinting while reading. Therefore, evaluating the efficacy of any presbyopic treatment should include studying its ability to improve functional reading at near distance.

Several patient-reported outcome (PRO) instruments have been utilized to measure the functioning aspects of living with presbyopia, including the National Eye Institute Visual Functioning Questionnaire-25 (NEI VFQ-25) [3, 4], National Eye Institute Refractive Error Quality of Life Instrument-42 (NEI RQL-42) [4, 5], and Near Activity Visual Questionnaire (NAVQ) [6]. However, none meet the standards in the FDA’s PRO Guidance due to inadequate documentation of content validity, insufficient psychometric measurement properties, and a lack of item content relevance to presbyopia symptoms and impacts [7–9].

This article describes the development of a new PRO instrument, the Near Vision Presbyopia Task-Based Questionnaire (NVPTQ), to assess near-distance reading ability, in accordance with the development standards described in the US Food and Drug Administration (FDA) PRO Guidance [7].

## Methods

Concept elicitation (CE) interviews were planned to inform the development of a new PRO instrument assessing near-vision reading ability. This study was conducted in accordance with the tenets of the Declaration of Helsinki, and all applicable local laws/regulations. Before study start, the Copernicus Group Independent Review Board reviewed and approved the qualitative study protocols, Quorum Independent Review Board reviewed and approved the clinical study protocols, and all patients provided written informed consent.

Concept elicitation interviews were conducted in-person with 20 participants with a clinically confirmed diagnosis of presbyopia. Semi-structured interviews were designed to explore and document the concepts relevant to presbyopia symptoms, impacts, coping behaviors, and treatment satisfaction that were most relevant and important to measure for presbyopic individuals. Inclusion and exclusion criteria were developed to ensure that the study sample was similar to the sample expected to be enrolled in presbyopia clinical trials. Specifically, subjects were required to be 40 years of age and older, have uncorrected visual acuity (VA) of 20/40 or worse for near vision, and have best corrected VA of 20/20 for distance vision. In an effort to achieve adequate participant representation of varying refractive errors, recruitment quotas included at least 6 myopic participants (> − 0.5D to < − 6.0D), at least 7 emmetropic participants (≤ − 0.5D to ≤  + 0.5D), 3 hyperopic participants (> + 0.5D to <  + 3.0D), and 7 participants with astigmatism (> 0.5D to < 3.0D). Recruitment targets for sex were 50% female and 50% male. Participants were recruited through 3 US-based sites, located in Bakersfield, CA, St. Louis, MO, and Newport Beach, CA. Trained and experienced qualitative researchers conducted the interviews using a semi-structured interview guide. Audio recordings of the interviews were transcribed verbatim and anonymized by removing identifying information such as names and places.

A coding scheme based on the research objectives was developed by the researchers prior to coding and was updated as necessary to reflect the actual terms subjects used to describe concepts, as well as to incorporate newly emerging data. The first transcript was coded by all team members and reviewed by the research manager to confirm intercoder reliability; all further transcripts were coded by one individual. The qualitative data were analyzed using a combination of grounded theory methods and traditional content analysis, and the data were assessed for conceptual saturation [10]. Upon completion of the interview analysis, an item-generation meeting (IGM) was held, during which the results of the CE interviews were reviewed and discussed. The IGM participants included measurement experts and sponsor representatives. To ensure that the questionnaire represented relevant concepts in presbyopia, an expert in optics provided input after selection of the initial concepts for measurement. After the IGM, a draft version of the NVPTQ was developed for further evaluation.

To test the relevance and interpretability of the draft items and to ensure the comprehensiveness of the instrument to the experience of individuals with presbyopia, cognitive debriefing (interviews were conducted with 20 participants with a clinically confirmed diagnosis of presbyopia). Interviews were conducted in person using a semi-structured interview guide and included a brief concept confirmation phase, followed by item-level debriefing. The inclusion and exclusion criteria were similar to those used for the CE interviews. Qualitative data were analyzed using traditional content analysis at the item and domain level. Findings were reviewed by the study team to inform whether revisions were needed for the instrument.

After the qualitative research, psychometric analyses were conducted for the NVPTQ using data from a Phase 2 multicenter, double-masked, randomized, vehicle-controlled, parallel-group study (NCT02780115). Study medication was administered once daily in the morning for 28 days. After screening, site visits occurred on Day 1 (Visit 1), Day 2 (Visit 2), Day 14 ± 2 (Visit 3), Day 21 ± 2 (Visit 4), and Day 28 ± 3 (Visit 5), with study medication administered at Hour 0 in-clinic. During site visits within the treatment period, Hour 1 was considered the peak-efficacy assessment time point, whereas Hour 8 was expected to be outside of the peak efficacy period. After the treatment period, all participants remained in the study for a 14-day follow-up period, during which site visits occurred on Day 1 (Visit 6), Day 7 ± 2 (Visit 7) and Day 14 ± 2 (Visit 8). The NEI VFQ-25 and the Patient Global Impression of Change (PGIC) were included in the study as concurrent PRO measures. The study included 151 participants in the modified intent-to-treat population, defined as all randomized participants with a baseline and at least 1 post-baseline assessment of mesopic, high-contrast uncorrected near visual acuity (UNVA).

Psychometric testing began with an item-level evaluation of response frequencies and inter-item relationships. To understand the structural relationship among the NVPTQ items, item response theory (IRT) was chosen rather than factor analysis due to the nominal nature of the data. Testlet variables were proposed to address local dependence between item pairs, and IRT methods were used to refine the way in which individual item scores were combined to form the testlet variables. Nominal response models were used to identify an ordering of response categories in the new testlet variables, and graded response models were used to evaluate the appropriateness of the proposed testlet definitions and the structural relationship among the NVPTQ items through examination of slopes and fit statistics.

After the IRT results were interpreted, a scoring algorithm was identified and the measurement properties of the resulting domain scores were assessed. Specifically, the reliability, validity, and responsiveness of the NVPTQ scores were evaluated, and thresholds for interpreting meaningful within-patient change were established. To confirm that the NVPTQ produces reliable scores, 2 types of reliability were evaluated. First, Cronbach’s coefficient α was calculated to assess the internal consistency of the NVPTQ scores [11]. If the coefficient α exceeds 0.70, then it is generally considered appropriate to combine the values together into a total score [12]. Second, test–retest reliability was computed using the intraclass correlation coefficient (ICC) for the NVPTQ scores between Day 21 Hour 1 and Day 28 Hour 1 using data from participants who were relatively stable on the PGIC (i.e., the same, slightly better, slightly worse) at the retest time point [13]. The ICC was computed using a 2-way mixed-effects regression model based on absolute agreement [14]. For ICCs, excellent reliability is indicated with an ICC > 0.9, good reliability is indicated by 0.75 < ICC ≤ 0.9, moderate reliability is indicated by 0.5 < ICC ≤ 0.75, and values below 0.5 indicate poor reliability [15].

To confirm that the NVPTQ produces valid scores, 2 methods for assessing construct validity were applied. First, correlations were produced between the NVPTQ scores and the NEI VFQ-25 to assess convergent validity (i.e., NVPTQ domain scores well-correlated with scores that measure similar concepts) and discriminant validity (i.e., NVPTQ domain scores less correlated with scores that measure dissimilar concepts). Second, known-groups methods were used to evaluate the construct validity of NVPTQ scores. Three groups were defined based on the mesopic high-contrast UNVA: 20/125 or worse; 20/80 and 20/100; and 20/63 or better. An η^2^ effect size was computed as the between-groups sum of squares divided by the total sum of squares. Values of 0.01 to < 0.06 are considered small, 0.06 to < 0.14 are considered medium, and 0.14 or larger are considered large [16].

To assess that the NVPTQ scores are able to detect changes over time, responsiveness methods were used to examine score changes. Participants were classified based on concurrent PRO measures at the same time points according to the following definitions:

- PGIC
  - Improved = complete improvement, far better, or moderately better
  - Not Improved = slightly better, no change, slightly worse, moderately worse, or far worse
- Mesopic high-contrast UNVA:
  - Improved = 3-line improvement or greater
  - Not Improved = worsening, no change, or less than a 3-line improvement.

Means and standard deviations (SDs) for the NVPTQ scores were reported for each of the change groups defined above. Guyatt's responsiveness statistic (GRS) was reported as an effect size comparing the improved group to the not improved group [17]. The GRS is computed as the mean change for the target group (i.e., improved) minus the mean of the change for the comparison group (i.e., not improved) divided by the SD of the comparison group (e.g., not improved) and is interpreted as small (0.2 to < 0.5), medium (0.5 to < 0.8), and large (≥ 0.8) [16]. Finally, methods highlighted in the FDA’s PRO Guidance for interpreting meaningful within-patient changes on the NVPTQ were considered, namely anchor-based methods and distribution-based methods [7, 18].

## Results

### Qualitative results

Of the 20 participants interviewed during concept elicitation (Tables 1, 2), the majority were female (*n* = 14, 70.0%), with an average age of 50.1 years (range: 41–57 years). The median and mode for near visual acuity in the right eye (OD), left eye (OS), and both eyes (OU) was 20/50. Emmetropia (*n* = 9, 45.0%) was the largest accommodative status of subjects, followed by myopia (*n* = 7, 35.0%) and then hyperopia (*n* = 4, 20.0%) and astigmatism (*n* = 4, 20.0%).

**Table 1:** Participants’ demographics

	Concept elicitation interviews (N = 20)	Cognitive debriefing interviews (N = 20)
Average age (SD), y	50.1 (4.7)	48.8 (5.0)
Range	41, 57	41, 59
Age category, n (%)		
40–44	3 (15.0)	5 (25.0)
45–49	3 (15.0)	6 (30.0)
50–54	10 (50.0)	6 (30.0)
55–59	3 (15.0)	3 (15.0)
Data unavailable	1 (5.0)^a^	0 (0.0)
Sex, n (%)		
Female	14 (70.0)	11 (55.0)
Male	6 (30.0)	9 (45.0)
Race, n (%)^b^		
White	9 (45.0)	14 (70.0)
Black or African American	4 (20.0)	2 (10.0)
Native Hawaiian or other Pacific Islander	2 (10.0)	0 (0.0)
Asian	1 (5.0)	0 (0.0)
American Indian or Alaska Native	0 (0.0)	1 (5.0)
Other	4 (20.0)^c^	3 (15.0)^d^
Ethnicity, n (%)		
Not Hispanic/Latino	15 (75.0)	16 (80.0)
Hispanic/Latino	5 (25.0)	4 (20.0)
Highest level of education, n (%)		
Some college or certificate program	8 (40.0)	10 (50.0)
College or university degree (2- or 4-year)	5 (25.0)	3 (15.0)
High school diploma/GED or less	4 (20.0)	7 (35.0)
Graduate degree	3 (15.0)	0 (0.0)
Work status, n (%)^b^		
Working full-time	13 (65.0)	12 (60.0)
Homemaker	3 (15.0)	2 (10.0)
Retired	3 (15.0)	2 (10.0)
Working part-time	1 (5.0)	3 (15.0)
Unemployed	0 (0.0)	2 (10.0)
On disability	1 (5.0)	0 (0.0)
Other	1 (5.0)^e^	0 (0.0)

GED, General Educational Development; SD, standard deviation; y, year

^a^The participant did not report his/her age

^b^Participants were able to select all that applied

^c^Other races reported were Hispanic/Spanish (n = 2, 10.0%), Mexican (n = 1, 5.0%), and Indian (n = 1, 5.0%)

^d^Other races reported were Hispanic/Spanish (n = 2, 10.0%) and Filipino (n = 1, 5.0%)

^e^Reported as “as the situation demands” (n = 1, 5.0%)

**Table 2:** Participants’ vision-related information

	Concept elicitation interviews (N = 20)		Cognitive debriefing interviews (N = 20)	
Uncorrected near-vision acuity in both eyes, n (%)^a^				
20/39 or better^a^	0 (0)		3 (15.0)	
20/40–20/59	14 (70.0)		11 (55.0)	
20/60–20/79	2 (10.0)		4 (20.0)	
20/80 or worse	3 (15.0)		2 (10.0)	
Data unavailable	1 (5.0)		0 (0)	
Uncorrected near-vision acuity in the right eyes, n (%)				
20/39 or better^b^	0 (0)		1 (5.0)	
20/40–20/59	13 (55.0)		12 (60.0)	
20/60–20/79	2 (10.0)		5 (25.0)	
20/80 or worse	4 (20.0)		2 (10.0)	
Data unavailable	1 (5.0)		0 (0)	
Uncorrected near-vision acuity in the left eyes, n (%)				
20/39 or better^b^	0 (0)		1 (5.0)	
20/40–20/59	14 (70.0)		11 (55.0)	
20/60–20/79	3 (15.0)		3 (15.0)	
20/80 or worse	2 (10.0)		5 (25.0)	
Data unavailable	1 (5.0)		0 (0)	
Average time (SD) with near-vision difficulty, y	7.6 (8.4)		7.5 (8.8)	
Range	1–36		0.5–37	
Near-vision treatment experience, n (%)^c^	Current	Past	Current	Past
Prescription reading glasses	10 (50.0)	3 (15.0)	13 (65.0)	1 (5.0)
OTC reading glasses	8 (40.0)	0 (0)	18 (90.0)	0 (0)
Magnifying glasses	9 (45.0)	1 (5.0)	2 (10.0)	0 (0)
Prescription glasses for distance vision	7 (35.0)	1 (5.0)	1 (5.0)	1 (5.0)
Prescription bifocals	5 (25.0)	1 (5.0)	3 (15.0)	0 (0)
Prescription progressive glasses	5 (25.0)	0 (0)	2 (10.0)	0 (0)
Contact lenses for near vision	0 (0)	2 (10.0)	0 (0)	1 (5.0)
Contact lenses for distance vision	1 (5.0)	1 (5.0)	0 (0)	0 (0)
No treatment	2 (10.0)	0 (0)	0 (0)	0 (0)
Prescription trifocals	0 (0)	0 (0)	1 (5)	0 (0)
Time (SD) using near-vision treatment, y				
OTC reading glasses	5.3 (1.9)		4.0 (4.0)	
Range	3.0, 8.0		0.1, 16.0	
n	6		18	
Prescription glasses	5.1 (6.7)		5.6 (6.2)	
Range	0.4, 20.0		0.6, 20.0	
n	8		12	
Prescription bifocals	3.5 (3.9)		7.0 (7.0)	
Range	0.1, 8.8		2.0, 15.0	
n	4		3	
Other	6.1 (7.9)		6.8 (7.4)	
Range	0.2, 15.0		1.5, 12.0	
n	3^d^		2^e^	
Time (SD) using distance-vision treatment, y				
Prescription glasses	11.2 (13.7)		1.5 (NA)	
Range	0.2, 41.4		NA	
n	9		1	
Prescription bifocals	4.3 (4.3)		8.5 (9.2)	
Range	0.1, 8.8		2.0, 15.0	
n	3		2	
Contact lenses	7.0 (NA)		0	
Range	NA		0	
n	1		0	
Other	0		1.0 (NA)	
Range	0		NA	
n	0		1^e^	

NA, not applicable; OTC, over-the-counter; SD, standard deviation; y, year

^a^Included 2 cognitive debriefing interview participants who had uncorrected visual acuity of 20/40 or worse in each eye separately (per the eligibility criteria), and 20/39 or better in both eyes. One cognitive debriefing interview participant had uncorrected visual acuity of 20/39 or better in both eyes and in each eye separately, and thus did not meet the eligibility criteria; however, this participant was included in analysis in error

^b^Refers to 1 of 2 participants with uncorrected visual acuity of 20/40 or worse in each eye separately (per the eligibility criteria), and 20/39 or better in both eyes

^c^Reflects all treatments that applied to each participant, as reported in their demographic information form and during their interview

^d^Included prescription progressive glasses (n = 2, 10.0%) and “target shooting” glasses (n = 1, 5.0%)

^e^Details were not collected during the cognitive debriefing interviews

The most frequently reported presbyopia symptoms were blurred near vision (*n* = 20, 100.0%) and eyestrain (*n* = 20, 100.0%); all reports of blurred near vision were spontaneous. The most frequently reported impacts pertained directly to reading different types of material: reading a restaurant menu (*n* = 20, 100.0%), reading a nutrition/recipe label (*n* = 19, 95.0%), reading on a cell phone/caller ID (*n* = 19, 95.0%), reading in low or dim lighting (*n* = 19, 95.0%), reading small print (*n* = 18, 90.0%), reading a book (*n* = 15, 75.0%), and reading newspapers (*n* = 11, 55.0%). Impacts with the highest average bothersome ratings, rated by at least 5 (25.0%) participants on a 0–10 scale (“0” being not bothersome at all and “10” being extremely bothersome), were reading books/newspapers/magazines and reading in low/dim light ($\overline{x }$= 7.5 and 6.9, respectively). Non-reading impacts were also reported, with feeling angry or frustrated (n = 17, 85.0%), forgetting glasses (n = 17, 85.0%), and relying on others to read materials (n = 14, 70.0%) being reported most frequently.

Saturation was considered to be achieved at the point when additional interviews were unlikely to yield new information (i.e., new concepts of importance and relevance to participants). Through a structured reporting process, concepts emerging from the interviews were analyzed for saturation in sets in the order the data were collected, specifically in 4 rounds of 5 interviews each. Through this assessment, it was established that conceptual saturation was achieved by the end of the third round of interviews, and the study sample size was deemed sufficient.

### NVPTQ content development

As difficulty with reading was the most frequently reported impact by participants during CE interviews, the ability to assess functional reading at near distance was determined to be an important and salient concept of interest to evaluate with the development of a new presbyopia PRO instrument. During the IGM, the team recommended creating a standardized performance-based PRO experiment that allows subjects to assess their own ability to perform near vision reading tasks typically performed on a daily basis. The tasks would be setup in clinic so that subjects would sit at a standardized distance away from the task, under standardized lighting conditions, read the task example, and answer questions about how well they were able to read text in the example. The task-oriented PRO would allow subjects to complete the reading task at their own pace and assess their vision-related reading ability and associated satisfaction in a subjective manner. Four paper-based reading tasks (that are typically completed on a daily basis) were then developed for the NVPTQ, which included excerpts from a book, newspaper article, nutrition label, and menu. Three exploratory electronic tasks were also developed, which included excerpts from text messages on an iPhone, book excerpt on iPad tablet, and a credit card statement on a Macbook Pro computer. The distance between the participant and the task material, and the text size of the task materials were all standardized to minimize compensatory behaviors used by participants. Lighting was also standardized at 250 lumens for photopic conditions, and 10 lumens for mesopic conditions. To control for learning effects in the tasks across repeated administrations, 8 comparable but different versions of each task’s reading material were generated so that they could be varied across administrations. While this new instrument would be performance-based, the NVPTQ would be considered a PRO rather than a performance outcome (i.e., PerfO) assessment because its resulting scores would be based on direct patient reports of their experience while performing the tasks rather than being based on their actual performance on the standardized tasks.

The draft NVPTQ included 21 items consisting of the 4 selected paper-based reading tasks, and 3 exploratory electronic tasks. For each task, participants were asked to read the specified material in both mesopic and photopic conditions, and then answer 3 questions that measured the following: (1) the participant’s self-reported ability to read the material; (2) the use of a coping behavior (i.e., squinting) to read the material; and (3) the participant’s self-reported satisfaction with his/her ability to read the material. The “squinting” item was included in the instrument to account for behavior that could not be standardized in the clinic setting. Response options for reading ability included: “I could not read any of the text due to problems seeing up close,” which ranged from “poor” to “excellent.” The item measuring squinting as a compensatory behavior included the response options: “No,” “Yes, and squinting helped me read the text,” and “Yes, but I could still not read the text.” Finally, response options for satisfaction with the ability to complete each task ranged from “very dissatisfied” to “very satisfied.”

After development of the draft NVPTQ tasks and items, 20 presbyopic individuals participated in the CD interviews to evaluate the instrument (Tables 1, 2). Seven participants reported their highest education level to be high school or less (*n* = 7, 35.0%); the remaining 14 participants reported completing at least some college or higher (*n* = 13, 65.0%). The median for near VA in the OD, OS, and OU was 20/50, 20/45, and 20/40, respectively. Since the inclusion/exclusion criteria were revised for the CD interviews to more accurately reflect the population that the sponsor was planning to recruit in their upcoming clinical trials, only emmetropes with presbyopia that required a + 1.00 to + 2.50 reading add were interviewed in this round. Overall, participants interpreted the NVPTQ paper-based tasks as intended and reported the reading samples included in the tasks to be relevant to their daily lives. Some minor revisions were made to the instructions based on participant feedback to ensure it was clear that the questions were asking about their ability to read the text, rather than their reading comprehension of the tasks. Additionally, the “squinting” response options were revised to include another option to account for participants who could squint but could only see “some of the text”. The exploratory electronic tasks were ultimately not included given the challenges associated with standardizing their appearance, as well as feedback from participants noting that the tasks did not accurately capture how they would interact with devices in daily life.

### Psychometric results

All 151 modified intent-to-treat participants completed all 12 NVPTQ items at Day 1 Hour 0, suggesting that there were no concerns with task administration or comprehension. At Day 1 Hour 0 (before treatment administration), NVPTQ item responses were skewed with the largest proportion of participants endorsing response categories towards the floor of the response scales (i.e., poor reading performance and dissatisfaction). For Performance, the most-frequently endorsed response category was the lowest category (i.e., no ability) for the book and newspaper tasks and the second lowest category (i.e., “poor”) for the menu and nutrition-label tasks, whereas for Satisfaction, all 4 tasks observed the lowest category (i.e., “very dissatisfied”) as the most-frequent endorsed. However, this skewed pattern was expected due to participants being symptomatic at Day 1 Hour 0. At this time point, squinting was used by approximately half of the participants on each task, and squinting was split between being helpful (range: 22–30%) and not helpful with reading (range: 15–26%).

Correlations between the 12 NVPTQ items at Day 1 Hour 0 showed a strong relationship between Performance and Satisfaction (range: 0.87–0.92, within task), whereas Squinting was observed to be moderately correlated with both Performance (range: − 0.28 to − 0.65, within task) and Satisfaction (range: − 0.34 to − 0.47, within task). Although the NVPTQ was developed with the intention of deriving a Performance domain score based on the responses to the 4 Performance items and a Satisfaction domain score based on the responses to the 4 Satisfaction items, this pattern of correlations suggests that the use of squinting may influence performance of and satisfaction with the reading tasks and, therefore, should be incorporated into the scoring algorithm.

Because the Squinting items correspond to the same tasks as the Performance and Satisfaction items, responses to these items cannot be considered locally independent, which is an assumption of IRT, the method selected to inform scoring. To account for this local dependence, the responses to the pairs of items for each task were reframed as a single testlet variable based on the cross-tabulation of all possible response categories (i.e., 18 possible response pairs for Performance and Squinting, 15 possible response pairs for Satisfaction and Squinting). However, while Day 28 Hour 1 was selected for IRT evaluation due to its observed variability across response categories, sparse cells in the cross-tabulations necessitated that testlet categories be collapsed before analysis (Table 3). The Performance and Squinting testlet variable was reduced to 10 categories, whereas the Satisfaction and Squinting testlet variable was reduced to 8 categories (Table 4).

**Table 3:** Item response frequencies for pairs of Performance + Squinting items and Satisfaction + Squinting items

Item Response Pairs	Book *n* (%)	Newspaper *n* (%)	Menu *n* (%)	Nutrition Label *n* (%)
Performance + Squinting				
“Yes, but I still could not read any of the text” and…				
“I could not read any of the text due to problems seeing up close”	18 (12.24)	15 (10.20)	9 (6.12)	10 (6.80)
“Poor”	11 (7.48)	9 (6.12)	4 (2.72)	14 (9.52)
“Fair”	0 (0.00)	0 (0.00)	1 (0.68)	2 (1.36)
“Good”	0 (0.00)	0 (0.00)	0 (0.00)	0 (0.00)
“Very Good”	0 (0.00)	0 (0.00)	0 (0.00)	0 (0.00)
“Excellent”	0 (0.00)	0 (0.00)	0 (0.00)	0 (0.00)
“Yes, and squinting helped me read some or all of the text” and…				
“I could not read any of the text due to problems seeing up close”	0 (0.00)	0 (0.00)	1 (0.68)	0 (0.00)
“Poor”	10 (6.80)	11 (7.48)	13 (8.84)	11 (7.48)
“Fair”	9 (6.12)	10 (6.80)	13 (8.84)	18 (12.24)
“Good”	1 (0.68)	2 (1.36)	4 (2.72)	5 (3.40)
“Very Good”	1 (0.68)	1 (0.68)	1 (0.68)	1 (0.68)
“Excellent”	0 (0.00)	0 (0.00)	0 (0.00)	0 (0.00)
“No, I did not squint” and…				
“I could not read any of the text due to problems seeing up close”	15 (10.20)	19 (12.93)	8 (5.44)	14 (9.52)
“Poor”	15 (10.20)	13 (8.84)	14 (9.52)	23 (15.65)
“Fair”	23 (15.65)	24 (16.33)	20 (13.61)	22 (14.97)
“Good”	22 (14.97)	21 (14.29)	32 (21.77)	12 (8.16)
“Very Good”	15 (10.20)	13 (8.84)	15 (10.20)	8 (5.44)
“Excellent”	7 (4.76)	9 (6.12)	12 (8.16)	7 (4.76)
Missing response to either item	0 (0.00)	0 (0.00)	0 (0.00)	0 (0.00)
Satisfaction + Squinting				
“Yes, but I still could not read any of the text” and…				
“Very dissatisfied”	20 (13.61)	16 (10.88)	9 (6.12)	12 (8.16)
“Dissatisfied”	7 (4.76)	8 (5.44)	3 (2.04)	11 (7.48)
“Neither satisfied nor dissatisfied”	2 (1.36)	0 (0.00)	2 (1.36)	2 (1.36)
“Satisfied”	0 (0.00)	0 (0.00)	0 (0.00)	1 (0.68)
“Very satisfied”	0 (0.00)	0 (0.00)	0 (0.00)	0 (0.00)
“Yes, and squinting helped me read some or all of the text” and…				
“Very dissatisfied”	3 (2.04)	4 (2.72)	7 (4.76)	4 (2.72)
“Dissatisfied”	8 (5.44)	6 (4.08)	11 (7.48)	15 (10.20)
“Neither satisfied nor dissatisfied”	7 (4.76)	9 (6.12)	9 (6.12)	10 (6.80)
“Satisfied”	3 (2.04)	5 (3.40)	5 (3.40)	6 (4.08)
“Very satisfied”	0 (0.00)	0 (0.00)	0 (0.00)	0 (0.00)
“No, I did not squint” and…				
“Very dissatisfied”	19 (12.93)	20 (13.61)	14 (9.52)	23 (15.65)
“Dissatisfied”	14 (9.52)	19 (12.93)	17 (11.56)	16 (10.88)
“Neither satisfied nor dissatisfied”	15 (10.20)	13 (8.84)	11 (7.48)	16 (10.88)
“Satisfied”	35 (23.81)	32 (21.77)	45 (30.61)	23 (15.65)
“Very satisfied”	14 (9.52)	15 (10.20)	14 (9.52)	8 (5.44)
Missing response to either item	0 (0.00)	0 (0.00)	0 (0.00)	0 (0.00)

Data are from Day 28 Hour 1

**Table 4:** Testlets variables for Performance + Squinting and Satisfaction + Squinting used for nominal IRT

	Squinting		
	Yes, but I still could not read any of the text	Yes, and squinting helped me read some or all of the text	No, I did not squint
Performance			
“I could not read any of the text due to problems seeing up close”	1	3	5
“Poor”	2	3	6
“Fair”	2	4	7
“Good”	2	4	8
“Very Good”	2	4	9
“Excellent”	2	4	10
Satisfaction			
“Very dissatisfied”	1	2	4
“Dissatisfied”	1	2	5
“Neither satisfied nor dissatisfied”	1	3	6
“Satisfied”	1	3	7
“Very satisfied”	1	3	8

**Fig. 1:**
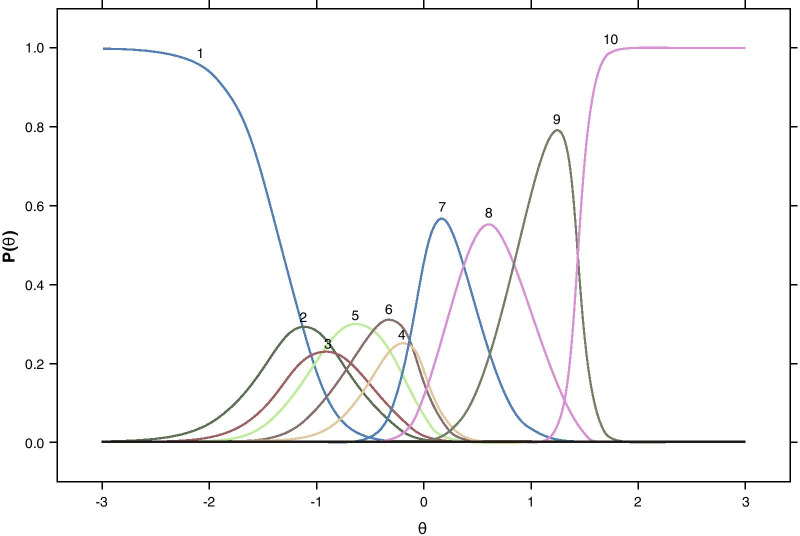
Nominal item characteristic curves for the Performance + Squinting testlet for reading a book. *Note* Curves are based on data from at Day 28 Hour 1 and correspond to the testlet responses as defined in Table 4

**Fig. 2:**
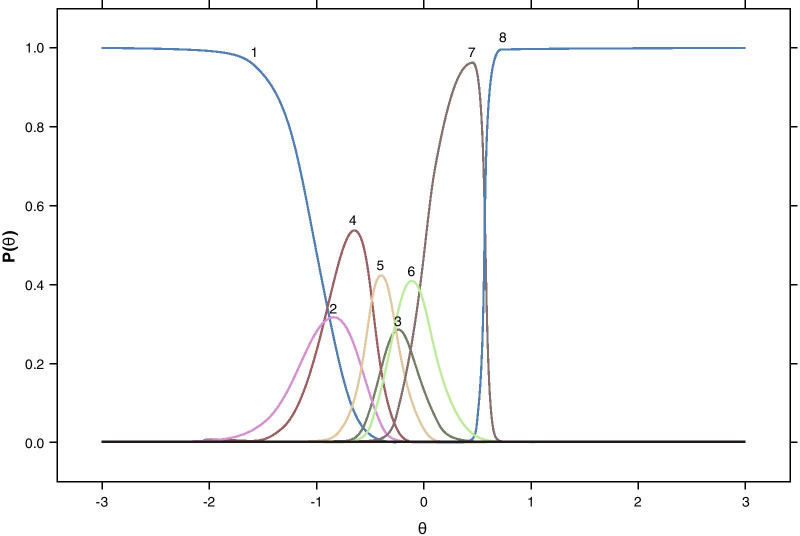
Nominal item characteristic curves for the Satisfaction + Squinting testlet for reading a book. *Note* Curves are based on data from at Day 28 Hour 1 and correspond to the testlet responses as defined in Table 4

As these testlet response categories do not have an inherent ordering (e.g., when considering the use of squinting that does not help in reading text compared with refraining from squinting and not being able to read the text), nominal response models were used to identify a linear order of the testlet categories so that a graded response model, which requires an a priori response category ordered, could be used to fully evaluate the NVPTQ items. An example item characteristic curve for Performance on the book task presented in Fig. 1 shows the pattern that was generally seen across all 4 tasks. The order of the Performance testlet category curves for the nominal model indicated that unhelpful squinting was associated with the poorest performance, and helpful squinting corresponded to lower performance than the non-squinting counterparts. For the Satisfaction testlet category curves (e.g., Fig. 2), the best testlet categories were clearly defined as satisfaction in the absence of squinting, whereas the worst testlet categories were observed as unhelpful squinting or helpful squinting with dissatisfaction, but the order for the remaining categories was more difficult to determine.

Based on the results of the nominal model, the Performance testlet response groupings were updated such that participants who did not squint when performing the task were assigned ordinal values ranging from the worst possible outcome (“I couldn’t read any of the text due to problems seeing up close” = 0) to the best possible outcome (“Excellent” = 5) (Table 5). Participants who reported helpful squinting had a single point deducted from their Performance responses (e.g., “Excellent” = 4). This is supported by the helpful squinting nominal response curves being shifted to the left of the non-squinting curves. Participants who reported unhelpful squinting were assigned a response value of 0 regardless of their performance rating because the Squinting item response indicates that they could not read any of the text, which is the worst possible outcome. A similar approach was taken for the Satisfaction response groupings so that an absence of squinting was assigned the original Satisfaction response scores (“Very dissatisfied” = 0, “Very satisfied” = 4), helpful squinting was assigned a numerical single step down (e.g., “Very satisfied” = 3), and unhelpful squinting was assigned the poorest outcome regardless of Satisfaction rating (Table 5).

**Table 5:** Testlets variables for Performance + Squinting and Satisfaction + Squinting used for graded IRT

	Squinting		
	Yes, but I still could not read any of the text	Yes, and squinting helped me read some or all of the text	No, I did not squint
Performance			
“I could not read any of the text due to problems seeing up close”	0	0	0
“Poor”	0	0	1
“Fair”	0	1	2
“Good”	0	2	3
“Very Good”	0	3	4
“Excellent”	0	4	5
Satisfaction			
“Very dissatisfied”	0	0	0
“Dissatisfied”	0	0	1
“Neither satisfied nor dissatisfied”	0	1	2
“Satisfied”	0	2	3
“Very satisfied”	0	3	4

**Fig. 3:**
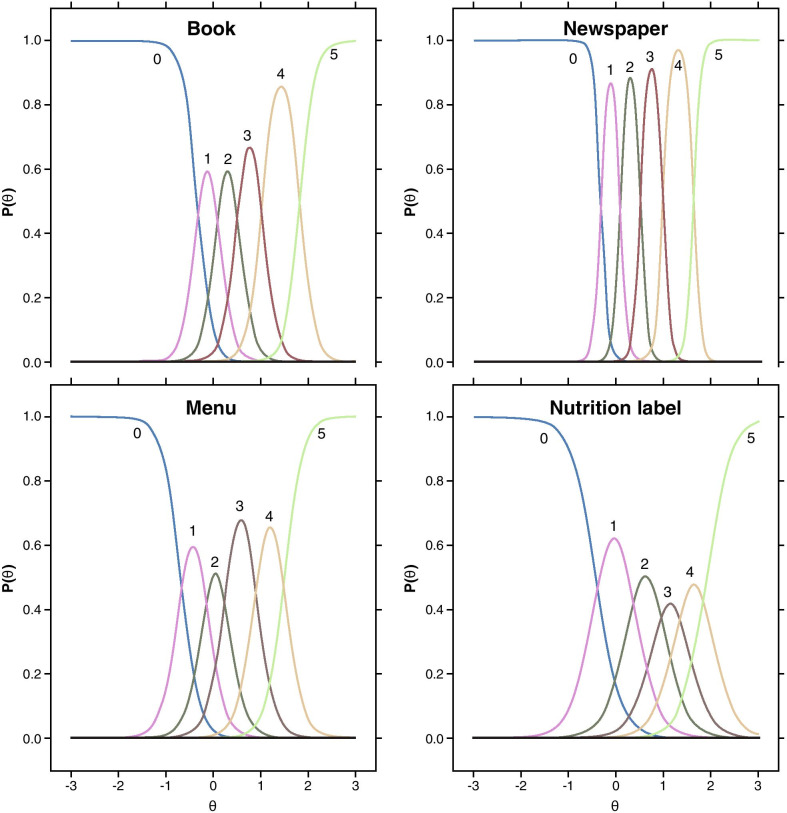
Graded item characteristic curves for the Performance + Squinting testlets. *Note* Curves are based on data from at Day 28 Hour 1 and correspond to the testlet responses as defined in Table 5

**Fig. 4:**
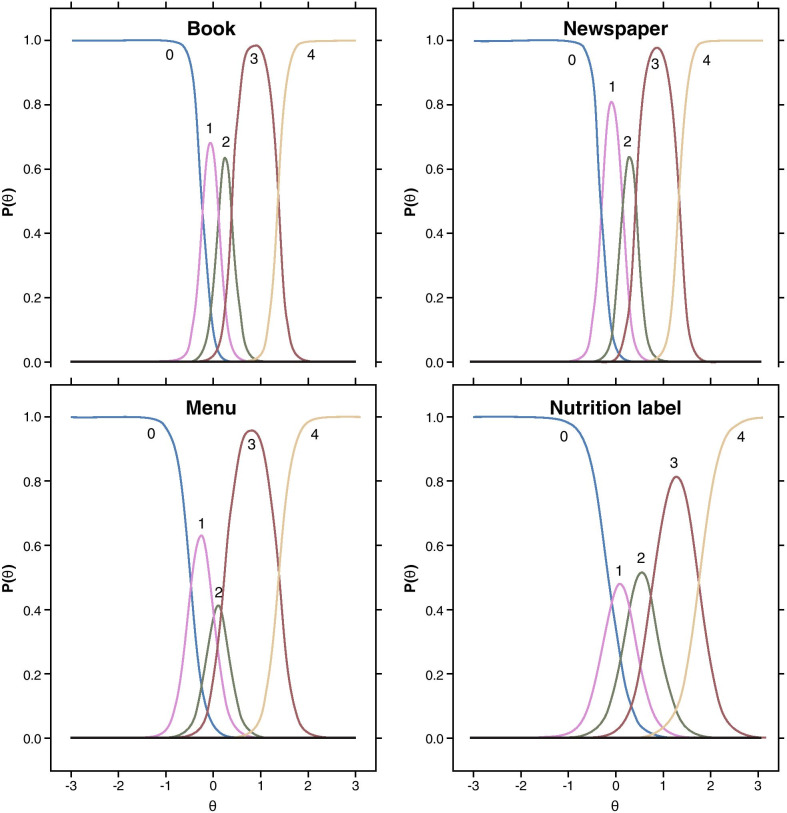
Graded item characteristic curves for the Satisfaction + Squinting testlets. *Note* Curves are based on data from at Day 28 Hour 1 and correspond to the testlet responses as defined in Table 5

The graded response model demonstrated that the 4 Performance tasks and 3 out of the 4 Satisfaction tasks each had good item fit, as determined by nonsignificant *S-X*^2^ values (*p* ≥ 0.05). Item characteristic curves for the Performance testlets and for the Satisfaction testlets are presented in Figs. 3 and 4, respectively (root mean square error of approximation [RMSEA] ≤ 0.05). Both the Performance model and the Satisfaction model had good fit with *pr(C*_2_*)* ≥ 0.05, RMSEA ≤ 0.05, standardized root mean square residual (SRMSR) ≤ 0.05, Tucker–Lewis index [TLI] ≥ 0.95, and comparative fit index (CFI) ≥ 0.95. Although these graded IRT results were supportive of the proposed testlet response ordering, the overall study sample size is modest for precise IRT parameter estimation, as evidenced by some unexpectedly large slopes among the items (Table 6). Further, some individual response category pairs (e.g., helpful squinting and excellent performance) did not have sufficient observations to allow for unique parameters to be estimated, thus making it difficult to generalize the IRT parameters derived here to scoring for future samples. Therefore, simple linear sum scoring based on testlets as supported by the IRT analyses was chosen for the NVPTQ scoring algorithm.

**Table 6:** Graded item parameters for the Performance + Squinting testlets and Satisfaction + Squinting testlets

NVPTQ Domain/Item Parameter	Book	Newspaper	Menu	Nutrition Label
Performance + Squinting				
* a*	6.40	13.04	5.24	3.86
*b*_1_	− 0.34	− 0.33	− 0.69	− 0.43
*b*_2_	0.09	0.08	− 0.17	0.32
*b*_3_	0.52	0.51	0.27	0.90
*b*_4_	1.02	0.98	0.90	1.36
*b*_5_	1.83	1.63	1.49	1.90
Satisfaction + Squinting				
*a*	10.12	10.24	6.66	4.70
*b*_1_	− 0.24	− 0.33	− 0.49	− 0.14
*b*_2_	0.09	0.12	− 0.04	0.30
*b*_3_	0.39	0.41	0.23	0.79
*b*_4_	1.36	1.30	1.38	1.76

Data are from Day 28 Hour 1

Testlets are scored according to the values in Table 5. In this scoring algorithm, participants who were helped by squinting are assigned one point lower than their observed score on the Performance or Satisfaction item to account for the fact that their vision-related reading ability and associated satisfaction likely received a small benefit from the act of squinting. Participants who could not read the text despite the use of squinting are assigned the lowest possible score to reflect that not being able to read the text is the poorest possible outcome on the scale. These testlet values are then used to compute domain scores as the average for the non-missing testlet values within the Performance domain, with scores ranging from 0 to 5, and within the Satisfaction domain, with scores ranging from 0 to 4. On both domains, higher scores correspond to better outcomes. If the response to either item within each testlet is missing, then the testlet is assigned a missing value. Because the 4 tasks are highly related, there is no limit on the number of missing testlet scores to be able to calculate the domain scores.

**Fig. 5:**
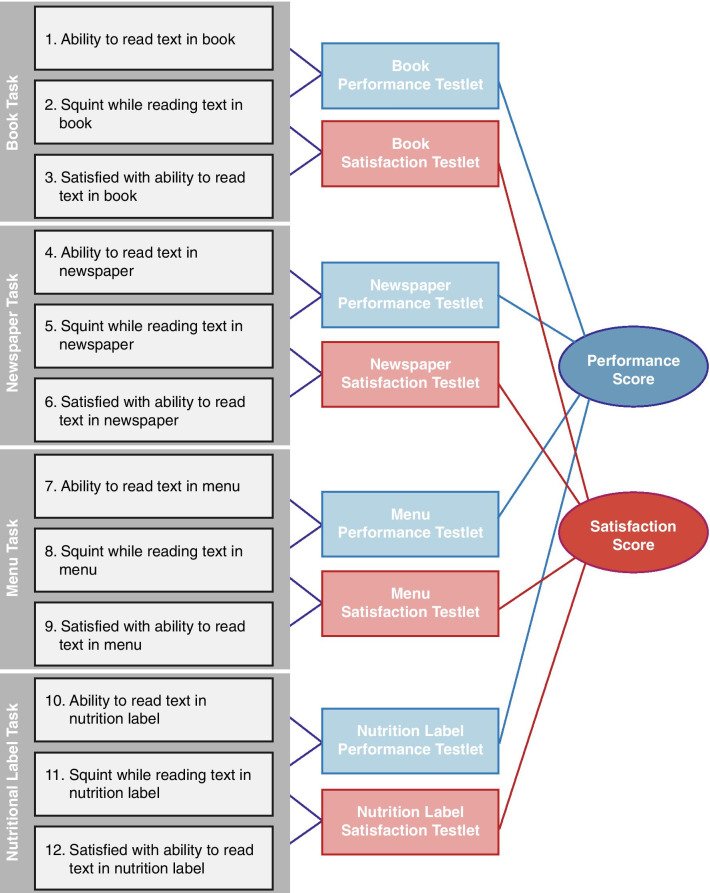
Near Vision Presbyopia Task-based Questionnaire Conceptual Framework. ©2021 AbbVie. All rights reserved

This NVPTQ scoring algorithm balances model fit with retention of concepts that are important to individuals with presbyopia. The conceptual framework (Fig. 5) displays the relationship between the 4 reading tasks and the 3 associated items (i.e., reading performance, reading satisfaction, and squinting) for each task.

The Cronbach’s α value for both NVPTQ domain scores at Day 28 Hour 1 far exceeded the recommended threshold of 0.70, with alpha of 0.96 for both the Performance and Satisfaction domain scores. This suggests that it is appropriate to combine the testlet values together into Performance and Satisfaction scores and the testlets are highly consistent with each other. No alpha-if-item-deleted values exceeded alpha, which indicates that no individual testlet is impairing the consistency of the domain score. All of the item-to-total correlations exceeded 0.40 and were generally similar within a domain, indicating that all testlets contribute similarly to the consistency of the scores.

The ICCs between Day 21 Hour 1 and Day 28 Hour 1 were 0.84 for Performance and 0.83 for Satisfaction. These values fall within the range classified as “good” test–retest reliability, so NVPTQ scores can be considered reliable over time.

Correlations were produced between the NVPTQ domain scores and the NEI VFQ-25 domain scores at Screening/Day 1 Hour 0 and at Day 21 Hour 1. The Pearson correlation with Near-vision Activities was expected to be strong (r ≥ 0.5) for both NVPTQ domain scores, but the correlations were only low to moderate in strength (range: 0.23 to 0.31 for Performance and 0.23 to 0.27 for Satisfaction). This pattern of correlations may be attributed to the NEI VFQ-25 instructing participants to answer as if they were wearing their glasses for correction, while the use of glasses during completion of the NVPTQ was not permitted. Across the full range of correlations, the Near-vision Activities domain had the strongest correlations with the NVPTQ domain scores at Day 21 Hour 0 (range of correlations for other NEI VFQ-25 domains: 0.01 to 0.28 for Performance and − 0.11 to 0.25 for Satisfaction), but the correlations at Day 21 Hour 1 exceeded Near-vision Activities for Mental Health with Performance (r = 0.31) and for Mental Health and Role Difficulties with Satisfaction (r = 0.33 and 0.26, respectively). Despite this unexpected trend, it is desirable to observe that Mental Health and Role Difficulties track with Performance and Satisfaction post-treatment.

NVPTQ domain scores were evaluated for 3 groups that were known to differ based on the clinical outcome of mesopic high-contrast UNVA at Day 1 Hour 0 and Day 28 Hour 1. Both Performance and Satisfaction scores at both time points were able to significantly distinguish between clinically differentiated levels of mesopic high-contrast UNVA (all *p* < 0.001). The poorest mesopic high-contrast UNVA values (i.e., 20/125 or worse) had the poorest scores on the Performance and Satisfaction domains, with mean values near 0. The best mesopic high-contrast UNVA values (i.e., 20/63 or better) had the best scores on the NVPTQ domains, with mean values of 1.44 and 1.96 on Performance at the 2 time points and mean values of 1.13 and 1.69 on Satisfaction at the 2 time points. The NVPTQ domain scores for the middle mesopic high-contrast UNVA group (i.e., 20/80 and 20/100) was consistently between the worst and best mesopic high-contrast UNVA group mean scores. The η^2^ effect sizes ranged from 0.14 for Satisfaction at Day 21 Hour 1 to 0.20 for Performance at Day 1 Hour 0, which are all considered “large.”

NVPTQ domain change scores were evaluated for groups defined as improved and not improved based on change on the patient outcome of PGIC and change on the clinical outcome of mesopic high-contrast UNVA from Day 1 Hour 0 to Day 28 Hour 1. Although the mean change for Performance and Satisfaction was positive for all improved and not improved groups, the Performance scores increased by 1.36 points on PGIC and 1.21 points on UNVA and the Satisfaction scores by 1.28 points on PGIC and 1.26 points on UNVA for the groups defined as improved, whereas the groups defined as not improved changed by no more than 0.58 points. The GRS effect sizes comparing improved and not improved groups were medium (GRS = 0.58 for UNVA) and large (GRS = 0.87 for PGIC) for Performance and medium for both Satisfaction groups (GRS = 0.67 for UNVA and 0.70 for PGIC).

Before proceeding with anchor-based analyses for interpreting change on the NVPTQ, the correlations between the anchors and the NVPTQ domain scores were reviewed to ensure that each anchor is viable for use in setting interpretation thresholds. The correlation with PGIC was 0.30 for Performance and 0.26 for Satisfaction, whereas the correlation with UNVA was 0.32 for both Performance and Satisfaction. Because the PGIC correlation with Satisfaction did not exceeded the a priori level of 0.30 for indicating a sufficiently related anchor, results for Satisfaction based on PGIC were reviewed, but given less consideration during triangulation.

**Fig. 6:**
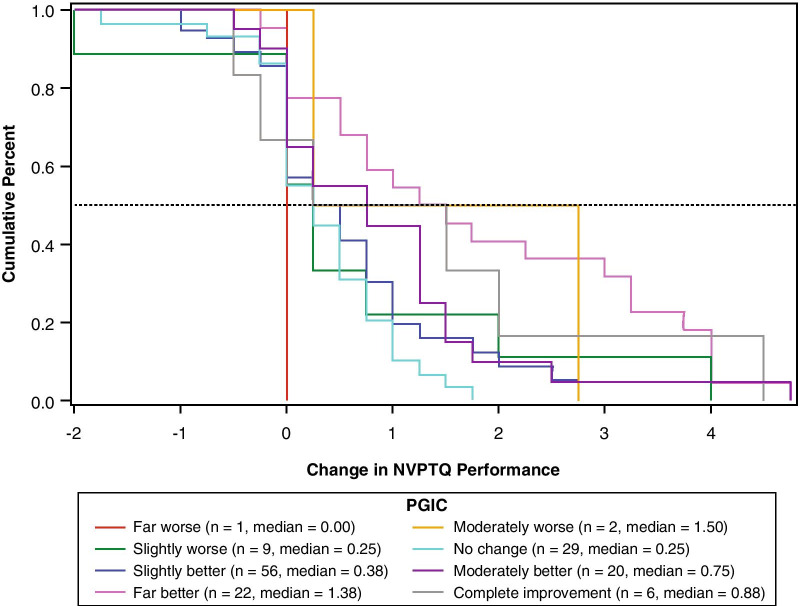
eCDF of change in NVPTQ Performance by PGIC. *Note* Change is computed as Day 28 Hour 1 minus Day 1 Hour 0. Positive scores indicate improvement. eCDF, empirical cumulative distribution; NVPTQ, Near Vision Presbyopia Task-based Questionnaire; PGIC, Patient Global Impression of Change

**Fig. 7:**
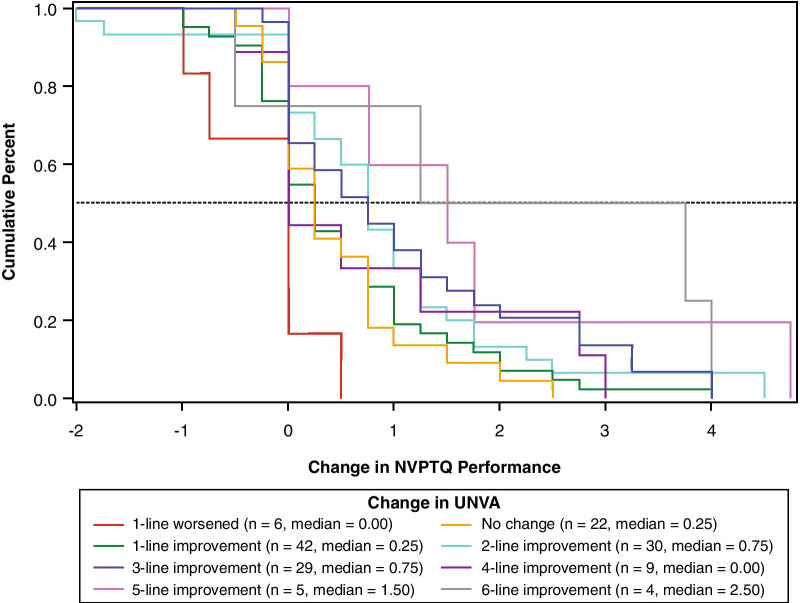
eCDF of change in NVPTQ Performance by change in UNVA. *Note* Change is computed as Day 28 Hour 1 minus Day 1 Hour 0. Positive scores indicate improvement. eCDF, empirical cumulative distribution; NVPTQ, Near Vision Presbyopia Task-based Questionnaire; UNVA, uncorrected near visual acuity

For each anchor and each NVPTQ domain score, empirical cumulative distribution functions (eCDFs), classification statistics (i.e., sensitivity, specificity, positive predictive value, negative predictive value), and nonparametric discriminant analysis were used to produce responder threshold estimates. A responder threshold is the change score at which an individual would need to meet or exceed to be classified as a treatment responder, which can be used for interpreting meaningful within-patient changes in a treatment setting. For NVPTQ Performance scores, which have a possible change score range from − 5 to + 5, the responder threshold estimates ranged from 0.6 to 2.6. Despite this range, the location where target anchor groups (i.e., moderately better on PGIC, 3-line improvement on UNVA) exceeds 50% on the eCDF was 0.75 for both PGIC (Fig. 6) and UNVA (Fig. 7). At this value, the classification statistics were well-balanced, while the discriminant analysis was less conclusive. Further, this threshold exceeds the distribution-based estimates of 0.41 for standard error of measurement (SEM) and 0.52 for one-half standard deviation (SD), so it can be considered sufficiently large to be reliably measured by the scale. Thus, the proposed NVPTQ Performance responder threshold is 0.75.

**Fig. 8:**
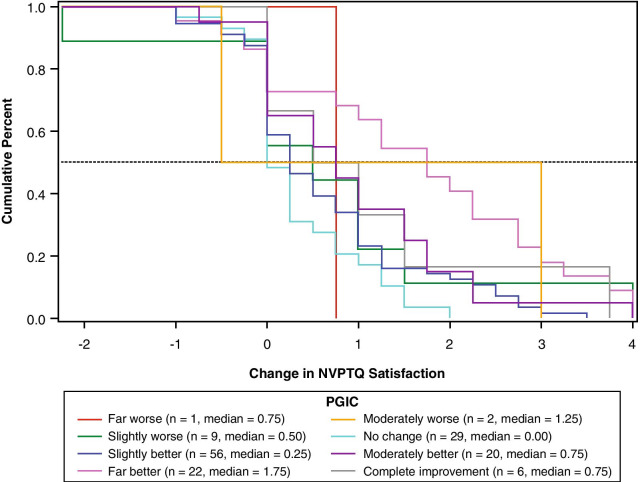
eCDF of change in NVPTQ Satisfaction by PGIC. *Note* Change is computed as Day 28 Hour 1 minus Day 1 Hour 0. Positive scores indicate improvement. eCDF, empirical cumulative distribution; NVPTQ, Near Vision Presbyopia Task-based Questionnaire; PGIC, Patient Global Impression of Change

**Fig. 9:**
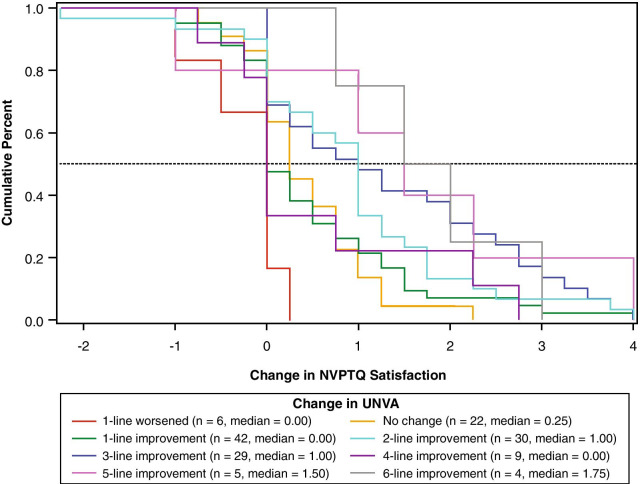
eCDF of change in NVPTQ Satisfaction by change in UNVA. *Note* Change is computed as Day 28 Hour 1 minus Day 1 Hour 0. Positive scores indicate improvement. eCDF, empirical cumulative distribution; NVPTQ, Near Vision Presbyopia Task-based Questionnaire; UNVA, uncorrected near visual acuity

For NVPTQ Satisfaction scores, which have a possible change score range from − 4 to + 4, the responder threshold estimates ranged from 0.6 to 3.6. Although the PGIC anchor also suggested a threshold of 0.75 on NVPTQ Satisfaction according to the eCDF results (Fig. 8), the PGIC was poorly correlated with NVPTQ Satisfaction. Thus, the UNVA eCDF estimate of 1.00 (Fig. 9) is a more appropriate threshold, and at this location the classification statistics were well-balanced (the discriminant analysis was less conclusive). This threshold also exceeds the distribution-based estimates of 0.40 for SEM and 0.49 for one-half SD, so it can be considered sufficiently large to be reliably measured by the scale. Therefore, the proposed NVPTQ Satisfaction responder threshold is 1.00.

## Discussion

The deterioration in near-vision acuity in individuals with presbyopia leads to difficulty performing essential near-vision tasks, such as reading [2]. As such, when assessing treatment benefit for individuals with presbyopia, it is critical to evaluate the impact on activities of daily life. To evaluate near-vision reading decrements from the patient perspective, the NVPTQ was developed based on a comprehensive and rigorous instrument development approach following the principles described in the FDA’s PRO Guidance [7]. The relevant patient-centered concepts were identified through a targeted literature review, and CE interviews confirmed the relevance of functional reading at near distance. Based on the types of reading activities for which CE interview participants reported having difficulty, a series of four paper-based reading-based tasks were constructed to measure vision-related reading ability and satisfaction with vision-related reading ability. CD interviews confirmed that these tasks and their associated patient-reported items were well-understood and relevant to individuals’ experience with presbyopia.

One limitation of the qualitative research is that the inclusion criteria for presbyopia participants differed between the concept elicitation and cognitive debriefing phases. While the concept elicitation phase included a broader range of participants (i.e., presbyopia participants that were emmetropic, myopic, hyperopic, and/or with astigmatism), the cognitive debriefing phase only included natural or surgery-corrected emmetropes at distance. Regardless, most participants in the cognitive debriefing phase still found the NVPTQ item content and tasks to be relevant to their experience. Another limitation is that the tasks included in the NVPTQ cannot account for or represent all of the potential activities that individuals with presbyopia complete on a daily basis. For example, it does not include non-reading activities (e.g., sewing, knitting, woodworking) that were reported as difficult to complete by presbyopia participants in concept elicitation interviews. These non-reading near vision tasks were ultimately excluded from the NVPTQ for the following reasons: Difficulty finding a non-reading task that was a common experience (e.g., putting on makeup, threading a needle were not tasks that all subjects had experience completing); difficulty of mimicking real-life non-reading tasks in a clinical setting (e.g., driving a car, repairing electronics, etc.); and difficulty with reading tasks were by far the most frequently reported impacts of presbyopia, as non-reading tasks impacts were less frequently reported, and instead could be assessed in a different PRO.

A Phase 2 psychometric evaluation of the NVPTQ provided support for the construction of Performance and Satisfaction scores that demonstrate strong psychometric properties and are easily interpretable. The NVPTQ Performance and Satisfaction scores are able to account for the impact of squinting in a simple and logical manner, resulting in a scoring algorithm that is parsimonious with face validity and is supported through extensive statistical testing using IRT.

The NVPTQ Performance and Satisfaction domain scores demonstrated strong internal consistency, good test–retest reliability, and desirable construct validity. The NVPTQ scores were further shown to be responsive to changes over time as defined by patient-reported and clinical variables. This pattern of psychometric properties supports the use of the NVPTQ in constructing presbyopia clinical trial endpoints. When interpreting results in a presbyopia clinical trial, score improvements of 0.75 points or greater on NVPTQ Performance domain and of 1.00 point or greater on NVPTQ Satisfaction domain may be considered clinically meaningful for an individual.

The totality of the evidence from the Phase 2 psychometric evaluation of the NVPTQ establishes a parsimonious and interpretable scoring algorithm and sufficiently robust measurement properties of the instrument scores. These psychometric properties were later confirmed in an independent Phase 3 clinical trial sample, providing additional support for the reliability, validity, and responsiveness of the NVPTQ scores, as well as the proposed score interpretation thresholds. This evidence supports the NVPTQ as an appropriate patient-centered endpoint to substantiate treatment efficacy claims for presbyopia clinical development programs. While the NVPTQ scores demonstrated good measurement properties, the modest sample size available from the phase 2 clinical trial is a limitation of this research. This sample of 151 is considered “very good” for reliability and validity (e.g., N ≥ 100) and “adequate” for responsiveness (i.e., 30–50 patients in the smallest group) according to the COSMIN Study Design checklist, but COSMIN considers samples less than 250 to be “inadequate” for multiparameter IRT models [19]. For this reason, we used IRT to inform the scoring algorithm, but we did not consider the IRT parameters stable enough for IRT scoring, opting for simple linear sum scoring instead.

The resulting NVPTQ is a novel type of clinical outcome assessment, combining assessment properties of a traditional PRO and a performance outcome measure. It is a traditional PRO in the sense that patients answer questions regarding their own ability to complete the reading tasks; however, it also incorporates aspects of a performance outcome as patients must complete specific tasks before answering questions. This novel approach strives to eliminate recall bias experienced by patients when answering the questions, while ensuring patients are responding to problems that they encounter in everyday life.

Although the other compensatory and coping behaviors during NVPTQ administration are controlled, a “squinting” item is included in the NVPTQ due to the inability to control for squinting when completing the tasks. Despite the inclusion of clear instructions, and an item that asks patients to assess the degree of squinting after completing each task, every effort was and should be taken to instruct patients to consciously minimize squinting during NVPTQ administration.

## Conclusions

The research conducted to develop and evaluate the NVPTQ has resulted in a content-valid and psychometrically sound instrument designed to evaluate vision-related reading ability and satisfaction with vision-related reading ability, which are important and relevant concepts to individuals with presbyopia.

## Data Availability

AbbVie is committed to responsible data sharing regarding the clinical trials we sponsor. This includes access to anonymized, individual and trial-level data (analysis data sets), as well as other information (eg, protocols and Clinical Study Reports), as long as the trials are not part of an ongoing or planned regulatory submission. This includes requests for clinical trial data for unlicensed products and indications. This clinical trial data can be requested by any qualified researchers who engage in rigorous, independent scientific research, and will be provided following review and approval of a research proposal and Statistical Analysis Plan (SAP) and execution of a Data Sharing Agreement (DSA). Data requests can be submitted at any time and the data will be accessible for 12 months, with possible extensions considered. For more information on the process, or to submit a request, visit the following link: https://www.abbvie.com/our-science/clinical-trials/clinical-trials-data-and-information-sharing/data-and-information-sharing-with-qualified-researchers.html.

## Abbreviations

CD: Cognitive debriefing
CE: Concept elicitation
CFI: Comparative fit index
eCDF: Empirical cumulative distribution functions
FDA: Food and Drug Administration
GRS: Guyatt’s responsiveness statistic
ICC: Intraclass correlation coefficient
IGM: Item-generation meeting
IRT: Item response theory
NEI VFQ-25: National Eye Institute Visual Functioning Questionnaire 25
NVPTQ: Near Vision Presbyopia Task-based Questionnaire
PGIC: Patient Global Impression of Change
PRO: Patient-reported outcome
RMSEA: Root mean square error of approximation
SD: Standard deviation
SEM: Standard error of measurement
SRMSR: Standardized root mean square residual
TLI: Tucker–Lewis index
UNVA: Uncorrected near visual acuity

## References

[1] Holden BA (2008). Global vision impairment due to uncorrected presbyopia. Arch Ophthalmol.

[2] McDonnell PJ, Mangione C, Lee P (2003). Responsiveness of the National Eye Institute Refractive Error Quality of Life instrument to surgical correction of refractive error. Ophthalmology.

[3] Mangione CM (2001). Development of the 25-list-item National Eye Institute Visual Function Questionnaire. Arch Ophthalmol.

[4] Berry S, Mangione CM, Lindblad AS (2003). Development of the National Eye Institute refractive error correction quality of life questionnaire. Ophthalmology.

[5] Hays RD, Mangione CM, Ellwein L (2003). Psychometric properties of the National Eye Institute-Refractive Error Quality of Life instrument. Ophthalmology.

[6] Buckhurst PJ, Wolffsohn JS, Gupta N (2012). Development of a questionnaire to assess the relative subjective benefits of presbyopia correction. J Cataract Refract Surg.

[7] US Food and Drug Administration (2009). Guidance for industry on patient-reported outcome measures: use in medical product development to support labeling claims. Fed Reg.

[8] Pesudovs K, Gothwal VK, Wright T (2010). Remediating serious flaws in the National Eye Institute Visual Function Questionnaire. J Cataract Refract Surg.

[9] McAlinden C, Skiadaresi E, Moore J (2011). Subscale assessment of the NEI-RQL-42 questionnaire with Rasch analysis. Investig Ophthalmol Vis Sci.

[10] Glaser BG, Strauss AL (1967). The discovery of grounded theory strategies for qualitative research.

[11] Cronbach LJ (1951). Coefficient alpha and the internal structure of tests. Psychometrika.

[12] Nunnally JC, Bernstein IH (1994). Psychometric theory.

[13] Shrout PE, Fleiss JL (1979). Intraclass correlations: uses in assessing rater reliability. Psychol Bull.

[14] Koo TK, Li MY (2016). A guideline of selecting and reporting intraclass correlation coefficients for reliability research. J Chiropr Med.

[15] Portney LG, Focratp WM (2000). Foundations of clinical research: applications to practice.

[16] Cohen J (1988). Statistical power analysis for the behavioral sciences.

[17] Guyatt G, Walter S, Norman G (1987). Measuring change over time: assessing the usefulness of evaluative instruments. J Chronic Dis.

[18] Coon CD, Cook KF (2017). Moving from significance to real-world meaning: methods for interpreting change in clinical outcome assessment scores. Qual Life Res.

[19] Mokkink LB, Prinsen CAC, Patrick DL et al (2019) COSMIN study design checklist for patient-reported outcome measurement instruments. https://www.cosmin.nl/wp-content/uploads/COSMIN-study-designing-checklist_final.pdf. Accessed 20 May 2021

